# IgM multiple myeloma presenting with spinal cord compression caused by a plasmacytoma: A case report

**DOI:** 10.1186/1757-1626-1-207

**Published:** 2008-10-02

**Authors:** Ihssan Tahan, Jim Seale, David Edwards

**Affiliations:** 1 Bone marrow transplant unit, Bristol Royal Hospital for Children, Upper maudlin street, Bristol, BS2 8BJ, UK; 2 Hematology Department, Northwest Wales NHS Trust, Ysbyty Gwynedd, Gwynedd, Banor, LL57 2PD, UK

## Abstract

**Background:**

IgM multiple myeloma is rare disorder, which has clinical, laboratory and radiological manifestations that are consistent with both multiple myeloma and Waldenstrom's macroglobulinaemia.

**Case presentation:**

An 83 years Welsh lady presented with clinical and radiological features consistent with spinal cord compression. Further investigations confirmed the diagnosis of IgM multiple myeloma. Following localized radiotherapy and five courses of melphalan and prednisolone, the patient achieved partial remission of her myeloma. Later on, the patient had disease progression in the form of rising serum IgM level and the development of multiple plasmacytomas. She was treated with thalidomide, cyclophosphamide, dexamethasone and radiotherapy, which resulted in the control of her disease for one year. To our knowledge, this is the second case of IgM myeloma presenting with a plasmacytoma and the first case of IgM myeloma presenting with cord compression caused by plasmacytomas.

**Conclusion:**

Unlike other types of multiple myeloma IgM myeloma is rarely complicated by plasmacytomas. However, spinal cord compression caused by plasmacytomas in this type of myeloma is extremely rare. Nevertheless, the same lines of management, e.g. cytotoxic chemotherapy and local radiotherapy that are applied to other types of myeloma can be successfully utilized.

## Background

Multiple myeloma (MM) is a B-cell lymphoid malignancy characterized by malignant clonal proliferation and accumulation of plasma cells that secrete paraproteins. Less than 1% of the cases are non-secretory. The incidence of this disorder is 3 per 100,000 per annum and the median age at diagnosis 60–65 years. In patients less than 40 years of age, the incidence of the disease is less than 2% [1,2]. The International Myeloma Working Group (IMWG) has reviewed the criteria for diagnosis and classification with the aim of producing simple, easily used definitions based on routinely available investigations. In monoclonal gammopathy of undetermined significance (MGUS), the monoclonal protein is < 30 g/l and the bone marrow clonal cells < 10% with no evidence of MM, other B-cell proliferative disorders or amyloidosis. In asymptomatic or smoldering myeloma, the M-protein is at least 30 g/l and/or the bone marrow clonal cells are at least 10% but there is no related organ tissue impairment or end-organ damage, which is typically manifested by increased calcium, renal insufficiency, anemia or bone lesions attributed to the plasma cell proliferative process. The diagnosis of symptomatic myeloma requires the presence of evidence of end-organ damage. However, non-secretory myeloma is characterized by the absence of M-protein in the serum and urine, bone marrow plasmacytosis and end organ-damage. Nevertheless, solitary plasmacytoma of bone, extramedullary plasmacytoma and multiple solitary plasmacytomas are still defined as distinct entities [1,2].

## Case presentation

**Figure 1:**
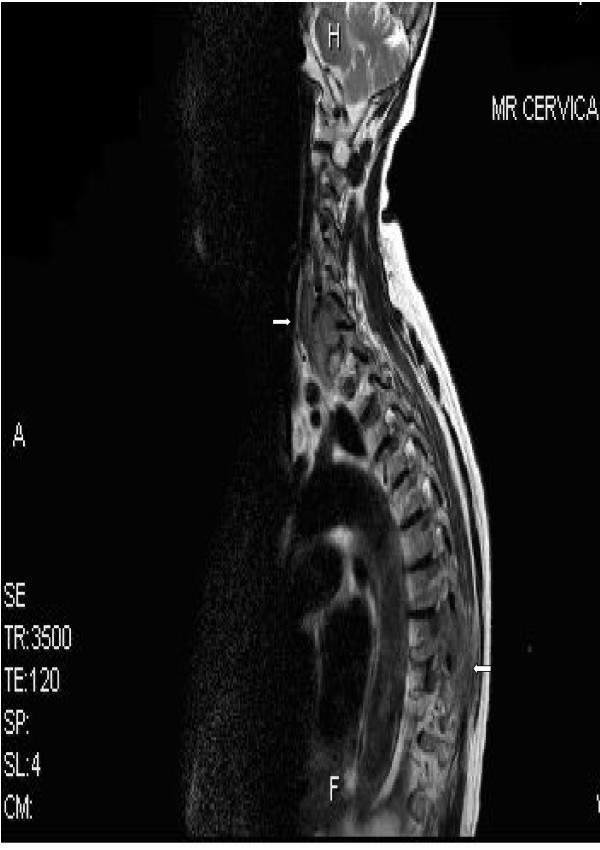
MRI scan shows two soft tissue masses compress the spinal cord at levels T8 and C6/C7.

**Figure 2:**
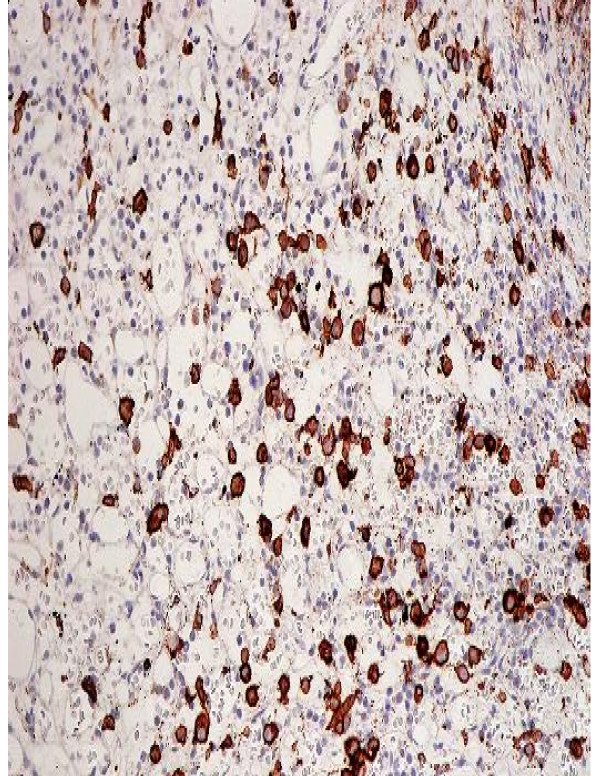
**Bone marrow Trephine biopsy showing CD38 positive cells {black-brown coloured}.** Both figures were taken at diagnosis.

An eighty three years old Welsh lady presented to the accident and emergency at Ysbyty Gwynedd Hospital in Wales with history of worsening weakness and tingling in the left upper arm for six weeks. Clinical examination showed brisk reflexes in the left upper limb, global weakness and increased tone in the same limb. An urgent MRI scan (Fig 1) revealed two soft tissue masses, one compressing the spinal cord at C6/C7 level and the other projecting into the cord and causing significant spinal cord compression at T8 level (Fig 1). Full blood count and biochemistry showed no abnormalities. Protein electrophoresis revealed monoclonal gammopathy with IgM paraprotein. Skeletal survey showed two lytic lesions. Bone marrow trephine biopsy showed infiltration with 20% of plasma cells. Immunohistochemistry revealed positive CD38 and negative CD20, CD19, CD56 and CD45 (Fig 2). A biopsy was not taken from the soft tissues described above due to technical difficulties. After establishing the diagnosis of IgM MM, the patient was commenced on radiotherapy, followed by five courses of melphalan and prednisolone. Disease progression was monitored by a series of protein electrophoresis assays, which showed a reduction in the paraprotein from 36 g/l at diagnosis to 6.5 g/l. The patient remained well for 7 months until a periumbilical mass 5 × 5 cm infiltrating the skin appeared. Consequently, biopsy of the lesion showed 56 percent infiltration by plasma cells. Computerized tomography scans revealed multiple masses having different sizes infiltrating kidneys and right breast. Then the patient was treated with CTD regimen (cyclophosphamide, thalidomide and dexamethasone) followed by palliative radiotherapy. Currently, the patient is still alive and well more than one year after receiving the last session of palliative radiotherapy.

## Discussion

MM syndromes have specific laboratory findings and are associated with monoclonal proliferation of plasma cells that usually secret monoclonal proteins, ultimately leading to bone destruction. Extramedullary tumors are not rare manifestations of MM. However, soft tissue is the commonest site involved. Having higher serum C-reactive protein and lactic dehydrogenase levels and the presence of a more advanced stage for international staging system (ISS), anemia and extramedullary tumors are the main poor prognostic factors of MM [3,4]. Waldenstrom's macroglobulinaemia (WM) is a B-cell lymphoproliferative disorder characterized primarily by the infiltration of lymphoplasmacytic cells into the bone marrow and the demonstration of IgM monoclonal gammopathy [5-8].

MM with IgM gammaglobulin is a rather distinct subtype of MM displaying clinical and pathologic features of both MM and WM [5]. It is a very rare disease that accounts for approximately 0.5 percent of all MMs. It usually presents with the same signs and symptoms as other types of myeloma and should be distinguished from WM based on clinical criteria, bone marrow morphology, immunophenotyping and cytogenetics as both disorders are different with respect to: modalities of treatment given, response to therapy and prognosis [5-8]. The clinical presentation of WM is similar to that of MM except that organomegaly is common in WM but is uncommon in MM and that lytic bony disease and renal disease are uncommon in WM but are common in MM [5-8]. So far, less than fifty cases of IgM myeloma have been reported. Ten percent of them had serum creatinine greater than 20 mg/l and serum calcium was greater than 120 mg/l in 24%. The mean serum IgM level was 33 g/l and the mean medullary plasmocytosis was 52%. Eighty per cent of the patients presented with IgM kappa and only 20% with IgM lambda. Proteinuria with light chains was found in 65% [5,9-12]. The cells in IgM myeloma appear to be arrested at a point of maturation between that of WM and MM. Preliminary data indicate that a pattern of osteoclast-activating factor and osteoprotegerin expression similar to that observed in classic MM is present in IgM myeloma [5,9-12]. Even though the cells of origin in WM and MM are mature and heavily mutated cells, they differ in IgH gene rearrangements. Especially in difficult cases of IgM MM, the search for t (11; 14) may be useful to discriminate them from WM [13,14]. The malignant cells in IgM myeloma have a distinctive chromosomal translocation that differentiates them from WM. These cells are post germinal-center in origin with isotype-switch transcripts. IgM myeloma is also characterized by negative CD20, D56 and CD117 phenotype and t(11;14) [13,14]. All the reported cases of IgM myeloma had been treated with melphalan and prednisolone with suboptimal results. Some patients were treated with VAD (vincristine, Adriamycin and dexamethasone) regimen, others with CTD protocol. The results were similar to the MP (melphalan and prednisolone) regimen. Radiotherapy was mainly used as palliative measure. Although elderly individuals are predominantly affected by the disease as other types of myeloma, young adults may rarely be affected. Eighty two per cent of patients survive for one year. The estimated survival is 82% for 1 year, 62% for 2 years and 46% for 3 years. However, no prognostic factor has been found.

## Conclusion

MM with IgM paraprotienaemia is a very rare disease, which can present as any other type of myeloma. It should be differentiated from WM. It is difficult to treat and the results of the treatment are usually sub-optimal.

## Competing interests

The authors declare that they have no competing interests.

## Authors' contributions

All the authors participated in the management of the patient presented.

## Consent

A written consent was obtained from the patient presented for the publication of the case report.
